# Budd–Chiari Syndrome Manifesting in Pregnancy: Case Report and Review of Management and Outcomes

**DOI:** 10.3390/reports9030259

**Published:** 2026-08-06

**Authors:** Hannah S. Foster, Gregory W. Kirschen, Sheri Bechard, Kristin D. Gerson

**Affiliations:** Department of Obstetrics and Gynecology, Hospital of the University of Pennsylvania, Philadelphia, PA 19146, USA

**Keywords:** Budd–Chiari syndrome, pregnancy, hypercoagulability, liver disease, fetal demise

## Abstract

**Background and Clinical Significance:** Budd–Chiari syndrome (BCS) is a rare disorder characterized by hepatic venous outflow obstruction, often associated with underlying hypercoagulable states. Pregnancy represents a physiologic prothrombotic condition that may precipitate disease onset. **Case Presentation:** We report a case of de novo BCS diagnosed in the second trimester in a previously healthy 36-year-old multiparous patient. Evaluation revealed cirrhotic liver morphology, portal hypertension, and bleeding esophageal varices requiring emergent treatment. The patient’s course included a transjugular intrahepatic portosystemic shunt (TIPS) procedure with complications, anticoagulation, multidisciplinary care, and a work-up revealing a JAK2 mutation consistent with an underlying myeloproliferative disorder. Despite apparent maternal stabilization and reassuring fetal growth, the pregnancy resulted in intrauterine fetal demise at 34 weeks’ due to placental abruption, followed by postpartum hemorrhage. **Conclusions:** This case highlights the diagnostic and therapeutic challenges of BCS in pregnancy, and demonstrates that favorable maternal stabilization does not preclude severe obstetric complications.

## 1. Introduction and Clinical Significance

Budd–Chiari syndrome (BCS) is a rare condition of hepatic venous outflow tract obstruction arising from thrombosis or primary pathology of the venous wall of the hepatic vasculature. It usually presents in the third and fourth decades of life, with slightly increased prevalence in women [1]. BCS may be indolent for a prolonged time period; however, known sequelae of BCS include portal hypertension, gastrointestinal varices, and liver-dysfunction-associated coagulopathy. Underlying hypercoagulable states are among the most important risk factors for the development of BCS. Given it may often present in an indolent, or chronic fashion, patients may present with subacute symptoms such as lower extremity edema, ascites, abdominal pain, or hepatomegaly.

Pregnancy is a physiologic hypercoagulable state that increases the risk of venous thromboembolism [2]. For individuals with an underlying predisposition to thrombosis, pregnancy may precipitate the development or clinical manifestation of BCS. Although BCS itself is not a contraindication to pregnancy, when the diagnosis is made in pregnancy, the potential risks and outcomes must be closely monitored [3,4]. While BCS in pregnancy is rare, it may be associated with inherited thrombophilias, myeloproliferative disorders, or other prothrombotic conditions. Typically BCS in pregnancy presents with abdominal pain, ascites, or elevated liver enzymes [5,6]. Adverse pregnancy outcomes associated with BCS include pregnancy loss and preterm birth, however based on the prior literature, outcomes are generally favorable [4,7,8,9,10].

Here, we present a case of acute BCS diagnosed in the second trimester of pregnancy in a patient without any known prior evidence of hepatic dysfunction. This patient had no prior known history of any risk factors for BCS and underwent a complex diagnostic and therapeutic course with eventual stabilization. However, despite her stable disease, she ultimately had an adverse obstetric outcome, highlighting the challenging and unpredictable nature of this condition.

## 2. Case Presentation

A 36-year-old previously healthy multiparous patient at 22 weeks’, 3 days’ gestation presented to a community-based medical center with new-onset hematemesis and acute abdominal pain. Her obstetric history was notable for one prior pregnancy of twins delivered at 37 weeks’. Her surgical history was notable for one prior cesarean delivery. She was taking no medications other than a prenatal vitamin. Her other pertinent histories were negative as was her review of systems.

In the emergency department, the patient had an episode of large-volume hematemesis. Her initial laboratory work-up was notable for anemia (hemoglobin of 8.4 mg/dL), hyperbilirubinemia (total bilirubin of 2.2 mg/dL), hyperammonemia (64 mg/dL), elevated INR (1.5), and normal transaminases. Initial computed tomography angiograph (CTA) of the abdomen and pelvis revealed cirrhotic morphology of the liver, splenomegaly, and evidence of esophageal varices. Abdominal ultrasound noted normal fetal heart rate.

The patient was intubated to protect her airway against aspiration, and an urgent endoscopy was performed. Findings were notable for grade 3 bleeding esophageal varices, most of which were successfully banded. She was then transferred to our tertiary care center for ongoing care. Portal hypertension and cirrhosis were diagnosed based on the combination of esophageal varices, CTA findings (portal hypertension, splenomegaly, gastric varices, splenorenal shunt, and pelvic ascites), and clinical presentation [11]. A broad work-up to evaluate her liver failure and associated portal hypertension was initiated. She was started on octreotide, a proton pump inhibitor (for ulcer prophylaxis after varices banding and risk–benefit analysis), and lactulose, and received five days of ceftriaxone for prophylaxis against spontaneous bacterial peritonitis [12]. Work-up for primary etiologies of cirrhosis was unremarkable, including negative family history, no personal history of alcohol use, no acetaminophen intake, a negative infectious hepatitis panel, negative autoimmune hepatitis serologies, negative cytomegalovirus antibody titers, negative Epstein–Barr virus antibody titers, and negative 24 h urine copper. The patient never had any prior hepatological evaluation such as examination of biomarkers of fibrosis, elastography, or thrombophilias. Due to concern for potential rebleeding, the patient was recommended to undergo transjugular intrahepatic portosystemic shunt (TIPS) procedure. She underwent an uncomplicated TIPS procedure at 23 weeks’ gestation, during which evidence of concurrent BCS was diagnosed, with partial hepatic vein occlusion. Postoperatively, she was continued on lactulose given her high risk of hepatic encephalopathy. She also initiated therapeutic anticoagulation with enoxaparin (1 milligram/kilogram), given the new diagnosis of Budd–Chiari syndrome. An abbreviated inherited thrombophilia work-up was obtained given her pregnant status, which included negative factor V Leiden and negative prothrombin gene mutation testing. A fetal growth assessment was performed at 24 weeks’ gestation, which revealed a normally grown fetus with an estimated fetal weight of 845 g. Throughout her admission, she received daily fetal heart rate assessments. She was discharged home on lactulose and enoxaparin. The remainder of her work-up was negative for antiphospholipid syndrome and both calreticulin and MPL genetic mutations (common mutations implicated in myeloproliferative disorders). Testing was positive for a JAK2 mutation.

The patient presented at 25 weeks’ gestation with recurrence of her initial symptoms and laboratory derangements, including hyperbilirubinemia and hyperammonemia. Initial imaging noted a patent TIPS, but suggested a hepatic artery pseudoaneurysm. Due to these findings, she subsequently underwent emergent embolization with interventional radiology. Her postoperative course was complicated by an episode of acute hepatic encephalopathy with ammonia that peaked at 365 mg/L. Repeat arteriography showed ongoing concern of bleeding at the pseudoaneurysm site, thus re-embolization was successfully performed. After extensive monitoring, the patient restarted therapeutic anticoagulation, which was monitored with anti-Xa levels. A postpartum bone marrow biopsy was recommended by hematology given her JAK2 mutation. The patient was discharged home at 26 weeks’, 3 days’ gestation. She was followed up with twice weekly antenatal testing beginning at 32 weeks’. The fetus remained well grown with a growth ultrasound at 31 weeks’ measuring 2002 g at the 60th percentile. She was scheduled to undergo repeat cesarean delivery at 37 weeks’ and zero days’.

**Figure 1:**
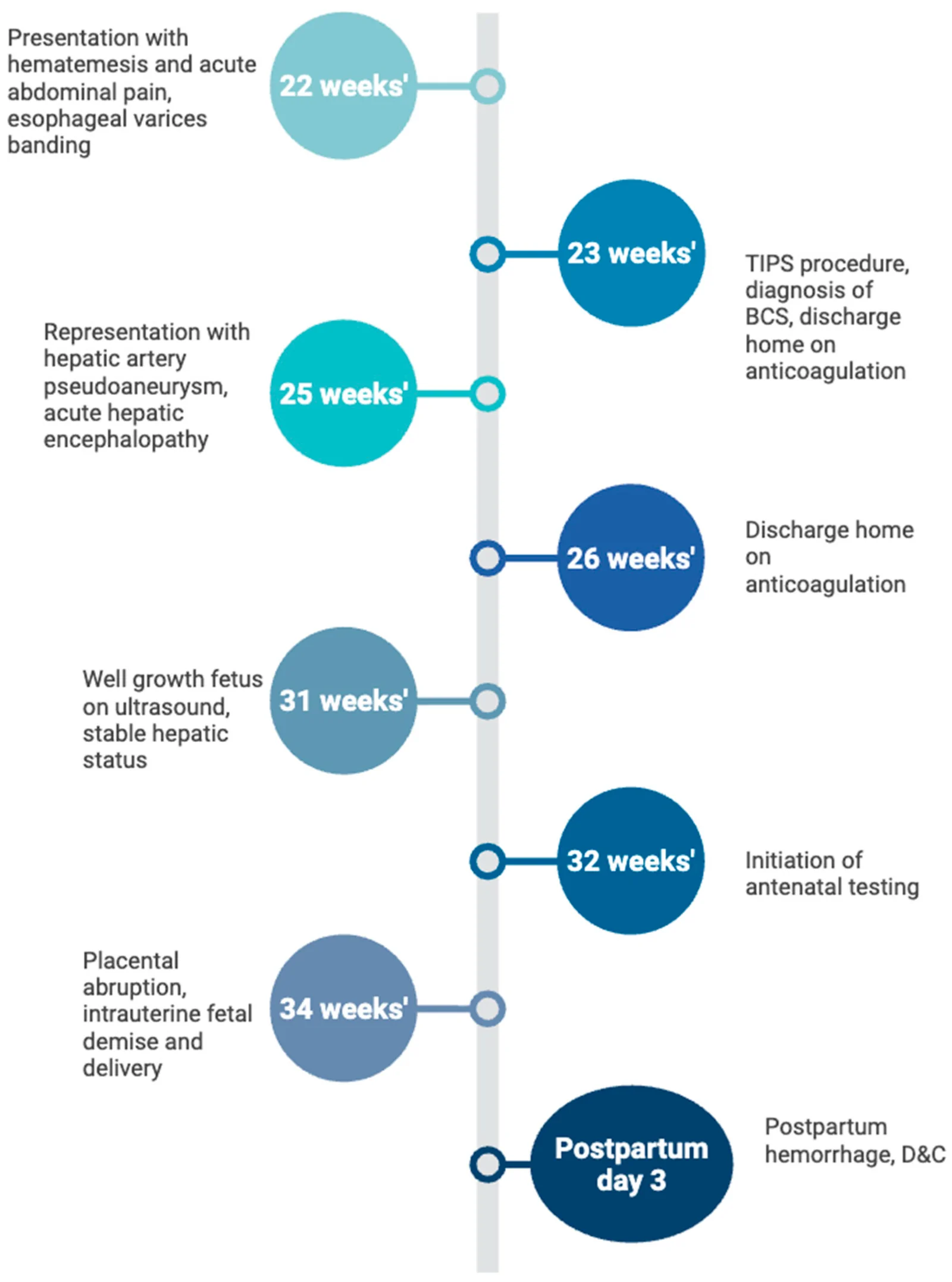
Timeline summarizing patient’s course.

At 34 weeks’ gestation, the patient began to experience severe pelvic pressure and abdominal pain followed by heavy vaginal bleeding. She presented for evaluation and was diagnosed with a fetal demise in the setting of placental abruption. She underwent induction of labor and had an uncomplicated vaginal delivery after cesarean. She was continued on therapeutic enoxaparin postpartum. On postpartum day three, the patient had recurrence of heavy vaginal bleeding and syncope, with a hemoglobin nadir to 6.5 mg/dL. She underwent emergent dilation and curettage for concern for retained products and received four units of packed red blood cells. A timeline depicting the patient’s course can be seen in Figure 1.

At the time of this case report, long-term follow-up for this patient is in process. The patient’s model for end-stage liver disease score was 18 as of five months postpartum, and her hepatology team is currently planning a follow-up endoscopy.

## 3. Discussion

This case highlights a novel presentation of de novo BCS in pregnancy, leading to the diagnosis of an underlying myeloproliferative disorder and a complex pregnancy. BCS is an uncommon but potentially life-threatening disorder, and the prior literature highlights pregnancy in patients with pre-existing BCS. Primary BCS is frequently linked to clonal hematologic disorders, particularly JAK2-positive myeloproliferative disease, even when overt hematologic abnormalities are absent at presentation [1]. In our patient, the eventual identification of a JAK2 mutation underscores this association and highlights the importance of comprehensive thrombophilia evaluation in cases of newly diagnosed BCS during pregnancy. Pregnancy itself represents a hypercoagulable state characterized by increased procoagulant factors, reduced fibrinolysis, and venous stasis. In patients with underlying hypercoagulable states, such as JAK2-associated myeloproliferative disease, this physiologic shift may precipitate hepatic venous thrombosis. JAK2-associated myeloproliferative disorders may specifically precipitate adverse pregnancy outcomes though placental dysfunction, leading to increased rates of fetal loss, fetal growth restriction, preeclampsia, and stillbirth [13]. A case report has described BCS diagnosed during pregnancy in the context of inherited thrombophilia, but large-scale data remain limited [14].

Maternal outcomes for patients with BCS are generally favorable when portal hypertension is well-controlled and anticoagulation is maintained. Most of the literature, including multicenter cohorts and case series, describes maternal and fetal outcomes in women with previously established and treated BCS rather than de novo diagnosis during pregnancy [2]. Studies suggest a relatively low rate of variceal bleeding, between 3 and 15% [4]. Cesarean delivery rates are as high as 70%, with cesarean delivery being standard in patients with esophageal varices [4,9]. Risk of maternal pulmonary hypertension as well as intrahepatic cholestasis of pregnancy also exist [9]. By contrast, acute presentation during pregnancy introduces diagnostic delay, limited imaging options, therapeutic complexity, and heightened maternal–fetal risk.

**Figure 2:**
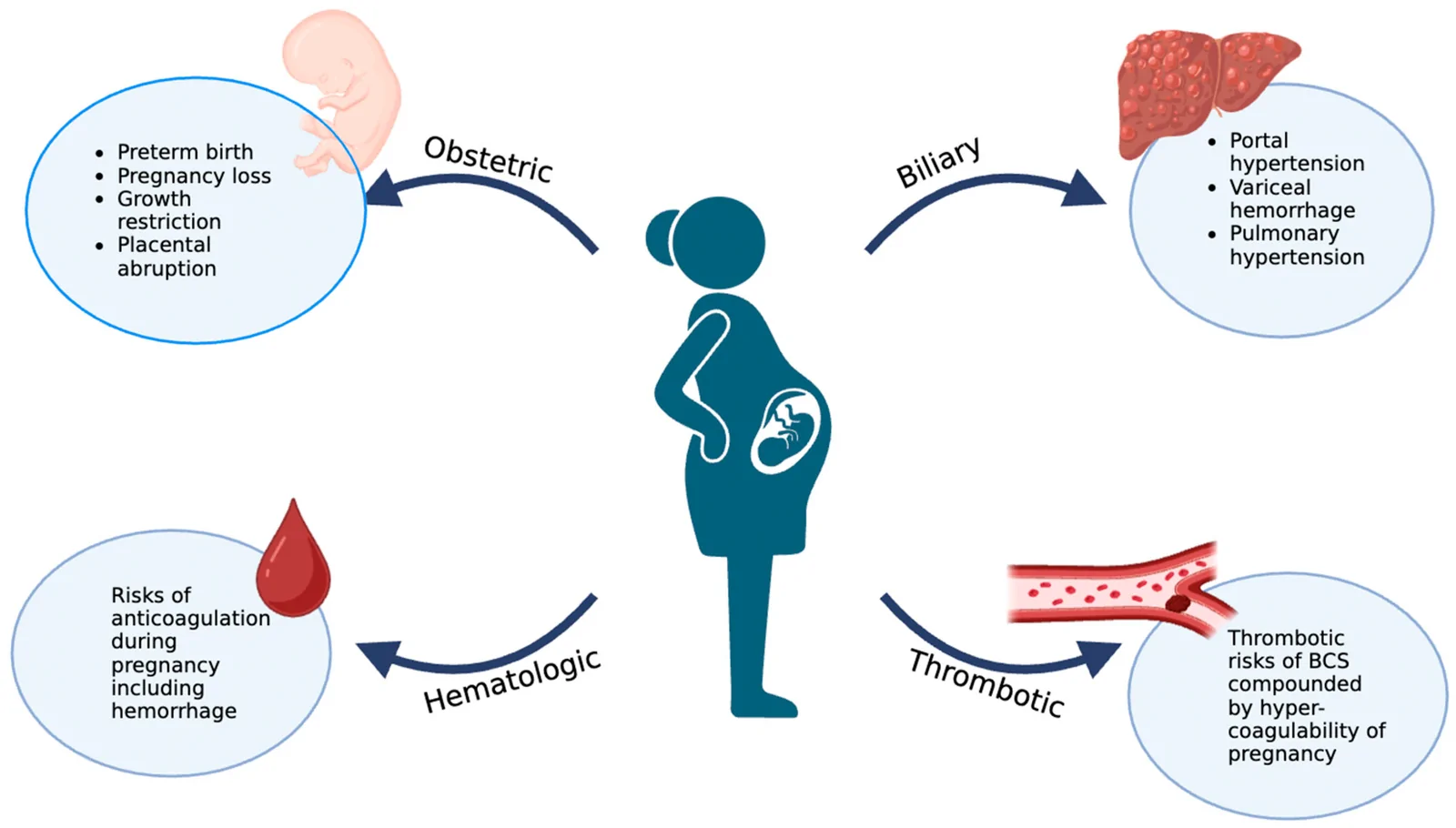
Cascade of concerns due to the BCS in pregnancy.

**Figure 3:**
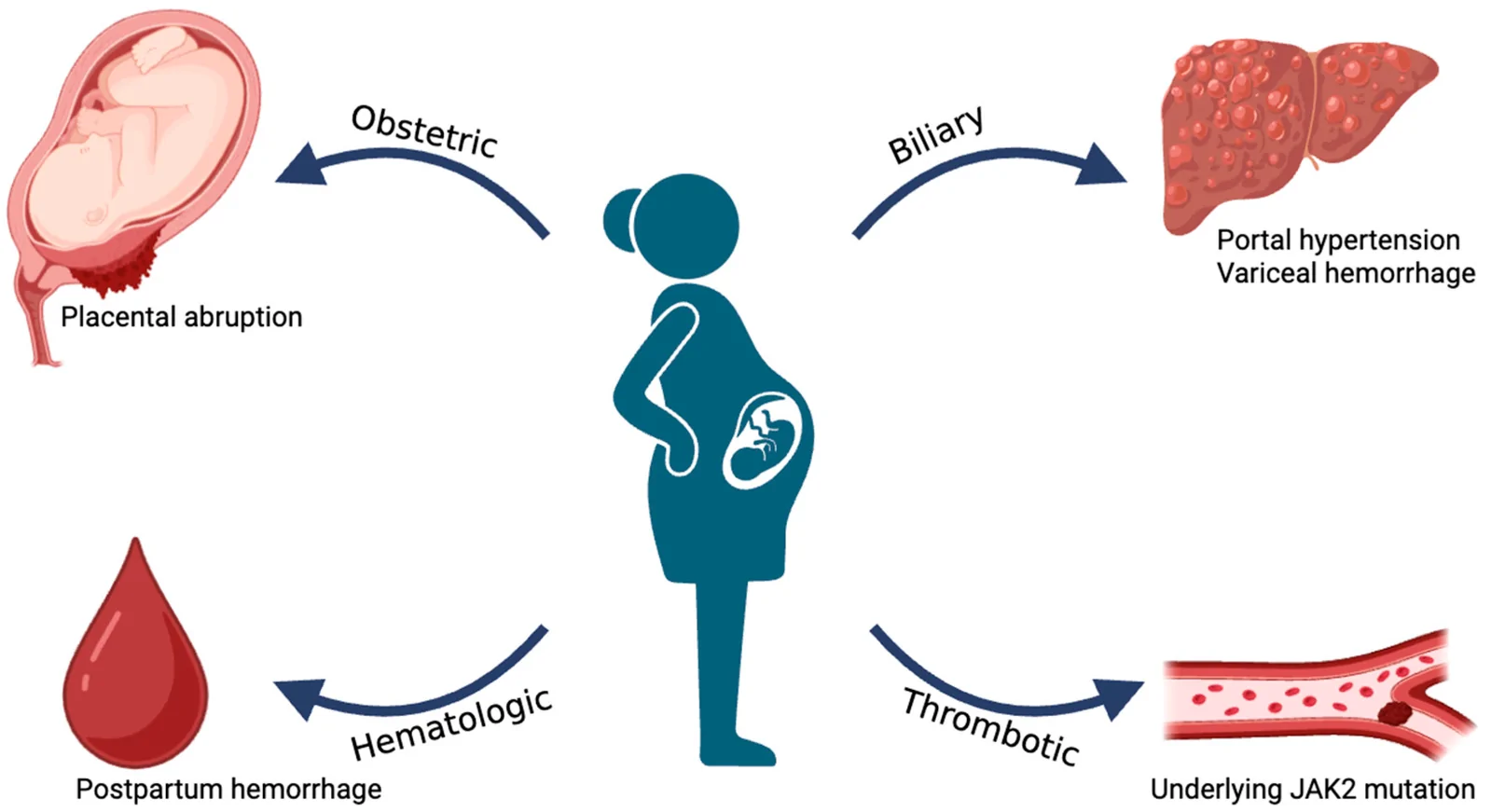
Cascade of concerns due to the BCS in pregnancy specific to this patient.

Fetal outcomes in women with known and treated BCS are generally favorable. Multicenter retrospective studies demonstrate acceptable live birth rates, though increased risks of preterm birth and pregnancy loss have been reported [3,4,9]. Risk of placental abruption and fetal growth restriction are also present [3,9]. A recent comprehensive review further notes that while many pregnancies progress without major complication under multidisciplinary care, fetal loss remains a documented risk, particularly in the setting of poorly controlled portal hypertension or thrombotic events [10]. Given this risk, despite inconsistent associations between thrombophilias and stillbirth or fetal growth restriction, antenatal fetal surveillance is warranted in these pregnancies [15]. These patients typically are at higher risk for placental thrombosis leading to insufficiency and fetal growth restriction or eventually stillbirth. Figure 2 highlights the cascade of concerns for BCS in pregnancy, whereas Figure 3 highlights the specific cascade of events for this patient.

Compared to the prior literature, this case is unique in that BCS was first diagnosed during the second trimester in a previously healthy patient without known liver disease, following a presentation with hematemesis due to grade 3 esophageal varices. Another distinguishing feature of this case is the severity of portal hypertensive complications at initial presentation. While the patient did not have any baseline liver function tests given her previously healthy state, her baseline pregnancy complete blood count was within normal limits. As mentioned, her initial imaging noted cirrhosis which does suggest a chronic process, perhaps exacerbated by the physiologic hepatic changes in pregnancy. Pregnancy increases splanchnic blood flow and intra-abdominal pressure, potentially exacerbating variceal risk, which may explain her acute presentation. The current literature supports endoscopic variceal banding and continuation of anticoagulation in pregnant patients with portal hypertension [9]. Our patient required emergent band ligation followed by TIPS placement. Although TIPS during pregnancy is rarely reported, available data suggest it can be performed successfully when maternal benefit outweighs fetal risk [16]. In this case, the procedure was technically successful; however, subsequent hepatic artery pseudoaneurysm and recurrent embolization highlight the procedural complexity and bleeding risk in anticoagulated pregnant patients with portal hypertension. Risk of pseudoaneurysm during a TIPS procedure is rare (<5%) [17]. Postoperatively, pregnancy physiology, specifically increased blood volume and cardiac output, may increase risk of hepatic encephalopathy [6,17].

In our case, serial fetal growth assessments and antenatal testing were reassuring. Despite apparent maternal stabilization and normal fetal growth, the pregnancy was complicated by intrauterine fetal demise at 34 weeks’ gestation secondary to placental abruption. The relationship between BCS and placental abruption is not well characterized; however, several mechanisms may be postulated. First, the prior literature has shown that underlying myeloproliferative disorders are associated with placental vascular pathology and adverse outcomes, including stillbirth [13]. Second, hepatic dysfunction and portal hypertension may theoretically contribute to altered maternal hemodynamics and coagulopathy, compounding placental instability [6]. Furthermore, theoretically splanchnic arterial vasodilation may lead to reduced circulating blood flow, resulting in hypotension and ultimately cirrhosis and portal hypertension. Similarly and finally, perhaps a similar etiology to intrahepatic cholestasis of pregnancy can be hypothesized, with hepatic dysfunction leading to elevated maternal bile acids, which may cause fetal and placental harm [18].

This case expands upon the existing literature by demonstrating that even with high-level multidisciplinary management including hepatology, maternal–fetal medicine, hematology, and interventional radiology, significant maternal and fetal morbidity from BCS may occur. It also highlights that reassuring fetal growth and antenatal testing do not eliminate the risk of devastating placental events in patients with systemic thrombotic disease. Antenatal testing has a negative predictive value of >99%; however, acute changes in fetal status, such as placental abruption, remain unpredictable. Lastly, postpartum hemorrhage further complicated this patient’s course, emphasizing the balance between thrombosis prevention with anticoagulation and hemorrhage risk. Therapeutic anticoagulation is a cornerstone of BCS management, yet peripartum anticoagulation requires coordination to minimize both thrombotic recurrence and bleeding complications.

## 4. Conclusions

In summary, de novo BCS presenting during pregnancy is rare and poses significant diagnostic and therapeutic challenges. The balance of hypercoagulability exacerbated by pregnancy must be closely balanced with risk of bleeding due to therapeutic anticoagulation, as The American Association for the Study of Liver Diseases suggests lifelong anticoagulation for BCS patients [19]. This case highlights the importance of maintaining a high index of suspicion for BCS in pregnant patients with acute hepatic failure and demonstrates the importance of early multidisciplinary management, prompt therapeutic anticoagulation, comprehensive thrombophilia evaluation, and close maternal and fetal surveillance. This report expands the limited literature on de novo BCS in pregnancy and provides practical insights to support timely diagnosis and management of similar cases.

## Data Availability

The original contributions presented in this study are included in the article. Further inquiries can be directed to the corresponding author.

## Abbreviations

The following abbreviations are used in this manuscript:

BCS	Budd–Chiari Syndrome
